# Monalizumab: inhibiting the novel immune checkpoint NKG2A

**DOI:** 10.1186/s40425-019-0761-3

**Published:** 2019-10-17

**Authors:** Thorbald van Hall, Pascale André, Amir Horowitz, Dan Fu Ruan, Linda Borst, Robert Zerbib, Emilie Narni-Mancinelli, Sjoerd H. van der Burg, Eric Vivier

**Affiliations:** 1 Department of Medical Oncology, Oncode Institute, Leiden University Medical Center, 2333 ZA Leiden, the Netherlands; 2 Innate Pharma Research Labs, Innate Pharma, Marseille, France; 3 Department of Oncological Sciences, Precision Immunology Institute, Tisch Cancer Institute, Icahn School of Medicine at Mount Sinai, New York, NY 10029 USA; 4 Aix Marseille Université, INSERM, CNRS, Centre d’Immunologie de Marseille-Luminy, Marseille, France; 5 Service d’Immunologie, Marseille Immunopole, Hôpital de la Timone, Assistance Publique-Hôpitaux de Marseille, Marseille, France

**Keywords:** Cancer immunotherapy, CD8 T cells, NK cells, NKG2A, Inhibitory immune receptor, HLA-E/Qa-1

## Abstract

The implementation of immune checkpoint inhibitors to the oncology clinic signified a new era in cancer treatment. After the first indication of melanoma, an increasing list of additional cancer types are now treated with immune system targeting antibodies to PD-1, PD-L1 and CTLA-4, alleviating inhibition signals on T cells. Recently, we published proof-of-concept results on a novel checkpoint inhibitor, NKG2A. This receptor is expressed on cytotoxic lymphocytes, including NK cells and subsets of activated CD8^+^ T cells. Blocking antibodies to NKG2A unleashed the reactivity of these effector cells resulting in tumor control in multiple mouse models and an early clinical trial. Monalizumab is inhibiting this checkpoint in human beings and future clinical trials will have to reveal its potency in combination with other cancer treatment options.

## Background

Immuno-oncology has emerged as a revolution in cancer treatment. Unprecedented improvements in tumor control have been achieved with therapeutic blocking antibodies that release immune inhibitory ‘checkpoints’ (immune checkpoint inhibitors, ICIs). In particular, therapeutic monoclonal antibodies (mAbs) directed against the PD-1 (programmed-cell death protein 1)/PD-L1 (programmed-cell death ligand 1) axis have been approved for use in monotherapy or combinations for several cancer indications [1–6]. Such treatments often yield sustained benefits, but strong responses are observed in only a minority of treated patients. Identification of predictive biomarkers for therapy response is subject of vigorous research at the moment and multiple factors have been determined. Among these factors are the number of T cells in the tumor and the total mutational load of tumor cells, indicating that ICIs depend on natural immunity targeting neoantigens presented by HLA molecules [7, 8]. Emerging lines of evidence also suggest that HLA class I genotype may predict tumor response to immune checkpoint blockade targeting PD-1 [9]. The studies found that maximal heterozygosity at *HLA-A*, *−B* and *-C* loci contributes to improved overall survival following ICI therapy compared to patients that were homozygous at one HLA class I locus with the largest effects at *HLA-B* and *-C* [9]. Primary or acquired resistance to ICIs is observed in a substantial fraction of patients [10], making it difficult to identify predictive markers of efficacy or recurrence. Major efforts are therefore being made to identify resistance mechanisms aiming to counteract tumor escape and thereby improve current therapies. Among those are anti-inflammatory cytokines (e.g. transforming growth factor (TGF)-β, IL-6 or IL-10 [11]), inhibitory metabolic factors (e.g. prostaglandin E2 [12, 13] and extracellular adenosine [14]), interferon signaling defects [15] and downregulation of classical HLA class I molecules [16], which are required for attack by tumor-specific cytotoxic CD8^+^ T lymphocytes. Loss of HLA class I expression on tumors is a well-established and common phenotype associated with many tumor types and has been linked to poor outcomes [16–25]. While the current understanding suggests that CD8^+^ T cells mediate the strongest anti-tumor response and that maximal heterozygosity is, by design, necessary to achieve optimal presentation of neoantigens, this narrative potentially underestimates the antitumor roles mediated by NK cells in response to ‘immuno-edited’ tumors. We recently reported that blockade of the immune checkpoint NKG2A recruits CD8^+^ T cell- as well as NK cell-reactivity to the stage [26, 27]. NKG2A is an inhibiting receptor expressed on subsets of cytotoxic lymphocytes and engages the non-classical molecule HLA-E [28, 29].

### Expression of the NKG2A ligands: HLA-E (human) and Qa-1 (mouse)

A view at the comprehensive tissue slide collection of the human protein atlas (www.proteinatlas.org) shows that HLA-E expression is, in general, ubiquitous but low. Exceptions are trophoblast cells in the placenta and ductal epithelial cells in the testis and epididymis, which display high levels of expression, suggesting a role for HLA-E in immune tolerance. Key factors of stabilization of the HLA-E protein at the cell surface are the availability of peptide ligands and proper function of the antigen processing machinery [30, 31]. Interestingly, the accommodated peptides are rather monomorphic and include those which derive from the leader sequences of classical HLA class I proteins (named ‘Qdm’ in the mouse and ‘VML9’ in humans). Maximal expression of *HLA-A*, *−B* and *-C* alleles on tumors promotes higher HLA-E cell-surface expression through provision of VML9 peptides [32], resulting in increased inhibition of NKG2A-expressing NK cells and CD8 T cells. All alleles of *HLA-A* encode a suitable HLA-E binding peptide, but polymorphisms across alleles drive differences in *HLA-A* expression [33, 34] and thus vary the amount of available HLA-E binding peptide [35]. Conversely, *HLA-B* is uniformly transcribed but has a dimorphism in its leader sequence at residue − 21 encoding either a good binding methionine (− 21 M) or a poor binding threonine (− 21 T) and thus varies whether or not it promotes HLA-E expression [36]. In mice, the inhibitory CD94/NKG2A receptor recognizes Qa-1 complexes with leader peptides from H-2D alleles. Both HLA-E and Qa-1 were crystallized and fold like conventional MHC class I molecules, but show strong preference for the Qdm/VML9 peptide [37, 38].

In contrast to classical HLA molecules which are frequently lost, HLA-E protein levels are generally increased in cancer when compared to their healthy counterparts, as described in lung, kidney, pancreas, stomach, colon, head and neck, liver, melanoma, prostate, and rectal tumor tissues [26, 39–41]. Exact mechanisms influencing this differential expression remain to be determined. However, anti-tumor immunity and IFN-γ, in particular, promote HLA-E expression at the tumor cell surface [42, 43]. The HLA-E-peptide complex is recognized by the CD94/NKG2A heterodimer receptor that is expressed by over 50% of either the CD56^bright^ immature or the CD56^dim^ mature NK cells from peripheral blood and on a subset of CD8^+^ T cells during chronic viral infections and in tumors [39, 44–46]. Engagement of CD94/NKG2A by HLA-E/Qa-1-expressing cells recruits the protein tyrosine phosphatase SHP-1 to the signaling synapse [47], resulting in the delivery of inhibitory signals to the effector cells and eventually inhibition of their immune activities [29, 43, 48]. NKG2A signaling appears to depend strictly on HLA-E/Qa-1 interactions and not on tonic signaling, since no detectable NK or T cell phenotype at steady-state has been observed [45, 49]. In head and neck, breast and non-small-cell lung cancer, invading NK cells express NKG2A [50, 51], and there is a correlation between high level of HLA-E expression and poor prognosis [39, 40, 52, 53]. Taking together, these observations strongly supported the scientific rationale for the generation of anti-NKG2A blocking antibodies aiming at unleashing the suppressive effect of NKG2A on NK and CD8^+^ T cell activity.

### Anti-NKG2A blocking therapeutic monoclonal antibody promotes both T and NK cell immunity

#### Blocking NKG2A signaling in mice releases both T and NK cell effector functions

**Fig. 1:**
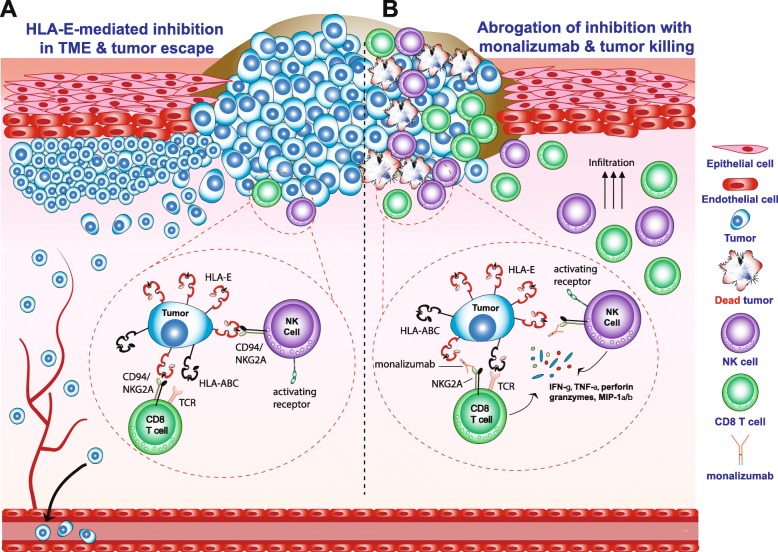
Schematic model describing the effects of HLA-E expression in the tumor microenvironment (TME) and the use of monalizumab to abrogate inhibition of NKG2A-expressing cells. **a** HLA-E expression on tumors mediates inhibition of NKG2A-expressing NK cells and CD8^+^ T cells and leads to tumor escape. **b** Use of NKG2A-blocking antibody monalizumab unleashes inhibition of NKG2A-expressing cells and promotes activation of NK cells and CD8^+^ T cells

Using a Qa-1^b+^ PD-L1^+^ A20 tumor model injected in BALB/c mice, in which both NK and CD8^+^ T cells are required to control tumor growth, almost half of the CD8^+^ tumor infiltrating lymphocytes (TILs) expressed PD-1 and importantly, half of them expressed NKG2A [27]. A majority of NK TILs expressed NKG2A, but PD-1 expression on NK cells was barely detectable. The tumor growth was controlled by combined blockade of NKG2A and the PD-1/PD-L1 (PD-x) axis, an effect that was dependent on both NK and CD8 T cells (Fig. 1). Moreover, the combined NKG2A and anti-PD-L1 blockade promoted tumor clearance in an additional mouse tumor model (RMA.Rae-1β) and favored the generation of protective anti-tumor memory CD8^+^ T cells that protected the hosts upon re-challenge with the same tumor.

#### Generation of monalizumab, a blocking anti-human NKG2A mAb, to liberate T and NK cell effector functions

In human cancer samples, HLA-E was demonstrated widely expressed on the surfaces of several tumor types. Therefore, NKG2A blockade, either alone or in combination with other checkpoint inhibitors, might improve the anti-tumor efficacy of NK and CD8^+^ TILs in cancer patients. Monalizumab, a humanized anti-NKG2A blocking mAb, increased degranulation and IFN-γ production by NKG2A^+^ NK cell against HLA-E^+^ target cells, thereby promoting NK cell effector functions [27]. It modestly increased the frequency of degranulating NKG2A^+^ Flu-specific-CD8 T cells upon restimulation with Flu-specific-peptide in vitro. Importantly, when used in combination with durvalumab, an anti-PD-L1 blocking mAb, monalizumab exhibited additive effects promoting both NKG2A^+^ PD-1^+^ NK and CD8^+^ T-cell effector functions. Also, when combined with cetuximab, an anti-epidermal growth factor receptor (EGF-R) mAb which promotes antibody-dependent cell-mediated cytotoxicity (ADCC), monalizumab enhanced the NK cell-mediated ADCC [27], suggesting that it would be interesting to investigate the effect of monalizumab to amplify the beneficial effects of other oncology treatments.

#### Use of monalizumab, a blocking anti-human NKG2A mAb, in combination with other oncoimmunology compounds to treat cancer patients

Following this rationale, evaluations of the efficacy and safety of monalizumab in cancer patients was conducted in phase II clinical trial using monalizumab in combination with cetuximab in patients with SCCHN (NCT026435509). In this interim report, an overall response rate (ORR) of 27.5% (95% CI 16–41%) was reported in 40 evaluable patients as compared to historical ORR of 13% observed for cetuximab monotherapy reported in earlier studies [27]. Monalizumab thus improved cetuximab response rates by unleashing NKG2A inhibition on lymphocytes, including NK cells. In this scenario, the mechanism of action of monalizumab likely consisted in the improvement of NK cell functions via antibody-dependent-cellular-cytotoxicity (ADCC) by the tumor targeting antibody cetuximab, and not by NKG2A-expressing CD8^+^ T cells.

Recently, dose escalation of first-in-human combination of monalizumab plus durvalumab in cohort of patients with metastatic microsatellite-stable colorectal cancer (MSS-CRC) has been completed (NCT02671435). Preliminary data demonstrate a manageable toxicity profile and indicate that the combination has encouraging activity in patients with MSS-CRC, a population historically nonresponsive to PD-1/PD-L1 blockade.

### Blocking NKG2A turns cancer vaccines into effective therapies

#### NKG2A is expressed on an unique CD8 T cell subset

In contrast to the rich literature of CD94/NKG2A receptors for NK cell biology, expression and function of NKG2A on adaptive immune cells is covered in paucity. In addition to NK cells, CD94/NKG2A is observed on subsets of innate lymphocytes, NKT cells, γδ T cells and CD8^+^ αβ T cells. The frequencies of NKG2A expressing CD8^+^ T cells in blood of SCCHN patients was very low, in the range of 2–10%, whereas up to 50% of NK cells expressed CD94/NKG2A [26]. Interestingly, frequencies in tumor infiltrating lymphocytes (TIL) were much higher for CD8^+^ T cells, indicating that NKG2A was induced in the tumor environment or that NKG2A-positive cells were selectively recruited there [26, 39, 46]. Previous literature suggested that T cell receptor triggering is required for induction of NKG2A and can be increased by IL-12 or TGFβ [54, 55]. CD8^+^ T cells recognizing tumor antigens indeed are more likely to display this inhibitory receptor [26]. CyTOF analysis of CD8^+^ TILs in cervical carcinoma samples interestingly suggested a preferential expression of NKG2A on T cells positive for the E-cadherin binding αEβ7 integrin. This CD103^+^ subset is associated with tissue residency, which is an epigenetically imprinted program mediating localization of lymphocytes to the tissues where they persist and patrol to protect organs for reoccurrence of pathogens [56]. The highly increased frequency of NKG2A in TIL versus blood CD8^+^ T cells and its higher expression on tissue resident cells versus other differentiation statuses of CD8^+^ T cells suggests an tissue-protective function for NKG2A on activated, antigen-specific lymphocytes [57–59]. However, whether these TILs represent real tissue-resident memory cells or active effector cells within tissues needs to be further unraveled. A recent study indeed report strong correlations between HLA-E expression in tumor lesions and frequencies of NKG2A^+^ CD8^+^ T cells [60]. To what extent this subset differs from those expressing PD-1 remains to be clarified in future studies. In any case, PD-1 expression seems more widespread on lymphocytes in cancers than NKG2A expression, which seems to be limited to tumor-attacking cytotoxic lymphocytes. Interestingly, frequencies of NKG2A expressing NK cells were rather comparable between blood and TIL and, moreover, between an immune reactive milieu induced by treatment and an immune silent milieu in untreated tumors [26]. NKG2A expression on other cytotoxic lymphocyte subsets, including type 1 innate lymphocytes (ILC1), NKT cells and γδ T cells, needs further investigation.

#### NKG2A blockade empowers anti-tumor CD8^+^ T cell immunity

NKG2A has been reported to regulate CD8^+^ T cell immunity to some viruses in that virus-driven immunopathology was limited and antiviral T cell responses were sustained by triggering NKG2A [44, 45, 61]. These mouse virus models implied a tempering role for overheated CD8^+^ T cell responses. In multiple cancer mouse models, NKG2A on CD8^+^ T cells functions as an immune checkpoint and blockade of the NKG2A/Qa-1 axis release the inhibitory signals (Fig. 1) [26]. In these models, CD8^+^ T cell immunity was induced by cancer vaccines, which were by themselves not strong enough to control tumor outgrowth. Pharmacological and genetic interruption of the NKG2A/Qa-1 interaction using blocking mAb and Qa-1 knockdown in tumor cells empowered these cancer vaccines and resulted in tumor regressions and durable clinical responses. These effects were not observed with NKG2A blockade alone, indicating a need for pre-existing antitumor CD8^+^ T cell immunity. Importantly, addition of PD-1 blockade instead of NKG2A blockade to cancer vaccines failed to improve survival of the mice, suggesting a differential role for these two checkpoints. The synergistic effect of NKG2A blocking antibody was demonstrated in four mouse tumor models and detailed analysis of the treated tumors revealed a strong increase of Qa-1 expression on tumor cells caused by T-cell derived IFN-γ and higher frequencies of NKG2A^+^ CD8^+^ T cells. Together, these pre-clinical data strongly instigate translation of this combinatorial treatment to cancer types for which off-the-shelf vaccines are available, like Human Papillomavirus (HPV) antigen comprising synthetic long peptide, RNA or DNA vaccines.

### Future perspectives

#### Critical involvement of NK cell responses for anti-tumor immunity

The importance of intratumoral CD8^+^ T cells for immunotherapy with checkpoint blockers is well recognized [7, 8], but more recently an indirect role of NK cells was revealed [62]. The NK cell frequency appeared to determine stimulatory dendritic cell numbers in the tumor and correlates with checkpoint responsiveness and increased survival. Mechanistically, production of the cytokine FLT3LG by NK cells defined this NK-DC axis [62]. Independent studies reached similar conclusions in that NK cell-mediated recruitment of conventional type 1 DCs (cDC1), which are BATF3 and CLEC9A positive, is essential for immunotherapy-responsive tumors [13, 63]. This type of immune-inflamed environment could be induced by TLR agonists, STAT1-activating signals and an anti-IL-10 antibody, leading to sensitization of tumors that displayed primary resistance to checkpoint blockade therapy [13, 63]. Importantly, several intervention strategies for the recruitment and activation of NK cells are emerging and will enable exploitation of these lymphocytes [64, 65]. Interestingly, cell cycle arrest and senescence, as induced by a combination of small kinase inhibitors, rendered tumor cells sensitive for NK cell attack and, moreover, another study recently revealed a role for the NKG2A-HLA-E axis in regulating immune-mediated clearance of senescent cells [66, 67]. Together, these studies indicate a plethora of opportunities to recruit NK cell immunity, and more specifically NKG2A blockade, into the field of cancer therapy.

#### Cancer vaccines might sensitize for NKG2A inhibition therapy

Although interest in cancer vaccines waned long ago due to a sheer lack of objective clinical responses in hundreds of trials, they recently regained attention since novel platforms demonstrated efficacy to induce broad CD4^+^ and CD8^+^ anti-tumor T cell immunity, increase immune infiltration of human cancers and eradicate pre-malignant lesions [68]. Recent clinical trials with cancer vaccines eliciting T cell immunity to personalized neoantigens or cancer virus antigens demonstrated promising prospects of this approach [69–71]. Moreover, vaccination therapy seems to combine very well with immune checkpoint blockade in that relapsed SCCHN patients responded well to a combination of nivolumab and a HPV16 peptide vaccine [70]. The addition of this long peptide vaccine improved the overall response rate and median overall survival. In the light of our recent findings on NKG2A, clinical trials with monalizumab and cancer vaccines are promising, but need to elucidate efficacy of this combinatorial approach.

#### HLA class I expression regulates both CD8^+^ T cells and NK cells in the tumor microenvironment

The human immune system relies on HLA class I to present antigens to CD8^+^ T cells while concurrently modulating NK cell inhibition and functional sensitization to tumors. Perhaps, the dual ability of HLA class I to regulate both NK cells and CD8^+^ T cells reflects differences in windows of immune activity, where NK cells lack the need for prior antigen-specific sensitization and can rapidly amplify the initial immune reaction [13, 62, 72–76]. Indeed, a recent study demonstrated increased NK cell infiltration in tumor regions of lung adenocarcinoma patients strongly associated with loss of heterozygosity (LOH) at the *HLA-C* locus compared to tumor regions without *HLA-C* LOH [77].

Analyses of genetic variation in *HLA-A*, *−B* and *-C* genes indicate that human populations are divided into groups that are stratified by HLA-E expression (higher threshold for NK cell activation) and the presence or absence of KIR ligands (degree of NK cell education) that define whether NKG2A-expressing or KIR-expressing NK cells are dominantly activated in response to cytokines, Fc-receptor-mediated signaling, and to loss of HLA-E or KIR ligands on tumors and HIV-infected CD4^+^ T cells [35, 78, 79]. Building on these emerging principles, a study of acute myeloid leukemia (AML) patients treated with IL-2 immunotherapy revealed patients with -21 M *HLA-B* alleles had significantly better leukemia-free and overall survival compared to patients that were homozygous for -21 T *HLA-B* alleles and found correlations with diminished expression of HLA-E on primary AML blasts [80].

Future studies should consider a comprehensive analysis of HLA class I expression and immunoediting of HLA genes in the germline and matched tumor tissues when considering alleles of HLA class I that are specifically lost (or even duplicated) and whether they promote high HLA-E expression and encode KIR ligands. The level of HLA-E expression and presence or absence of KIR ligands in germline tissue will determine the educational environment and subsets of NK cells that are trained to react to perturbed expression of HLA on tumors, which has been shown to vary extensively across cancers [81].

#### CMV reactivation and adaptive NK cells in the tumor microenvironment

Understanding the effects of cytomegalovirus (CMV) infection (and reactivation) is also important in settings of cancer immunotherapy for its ability to imprint NK cell phenotypes and functions and promote expansion of adaptive or “memory-like” NK cell subsets (range: 0–70% of the total circulating NK cells) [82]. Such expansions of adaptive NK cells have been observed in approximately 40% of healthy, latently infected individuals. In CMV infected individuals, adaptive NK cells have enhanced capacities for antibody-dependent cellular cytotoxicity (ADCC) and are particularly responsive to modulation of HLA-C on the surface of tumor cells. In most instances, CMV infection and adaptive NK cells are established well before tumorigenesis. Thus, CMV infection and adaptive NK cells may play an unappreciated role in potentiating ADCC reactivity to antibodies targeting tumor antigens (and to auto-antibodies, potentially contributing to treatment-related autoimmune toxicities). Intriguingly, higher HLA-E expression may be preferred for exploiting adaptive NK cell functions for immunotherapies. Adaptive NK cells preferentially express the activating isoform of NKG2A, NKG2C, and its recognition of HLA-E elicits an activating signal. Adaptive NK cells also express self-KIR2DL receptors making them particularly poised for recognizing HLA-C. Thus, somewhat counter-intuitively, CMV seropositive patients with high cell-surface expression of HLA-E may experience added protection from expanding adaptive NK cells where the therapeutic mechanisms of action are aimed at ADCC or abrogating inhibition through HLA-C, e.g. with lirilumab.

## Conclusion

NKG2A^+^ NK cells represent over 50% of peripheral blood NK cells and is also expressed on a subset of activated CD8^+^ T cells during chronic viral infections, such as human immunodeficiency virus (HIV) [35] and hepatitis C-virus (HCV) [83], and in tumors [26]. It is unclear why large proportions of CD4^+^ T cells remain NKG2A negative. André and colleagues showed that monalizumab can potentiate other ICI in a combination therapy, such as anti-PD-1/PD-L1 [27] and Van Montfoort and colleagues demonstrated efficacy in combination with cancer vaccines [26]. A central paradigm in current oncoimmunology is ‘*combinations’* and future clinical trials will need to carefully determine which combination therapy provides the best results in the interest of our patients.

## Data Availability

Not applicable

## Abbreviations

ADCC: Antibody dependent cellular cytotoxicity
AML: Acute myeloid leukemia
CMV: Cytomegalo Virus
CTLA-4: Cytotoxic T-Lymphocyte Associated Antigen 4
CyTOF: Mass Cytometry by Time-of-Flight
HCV: Hepatitis C Virus
HIV: Human immunodeficiency virus
HLA: Human leukocyte antigen
HPV: Human papilloma virus
ICI: Immune checkpoint inhibitor
IFN-γ: Interferon γ
KIR: Killer cell Immunoglobulin Receptor
LOH: Loss of Heterozygosity
mAb: monoclonal Antibody
ORR: Overall response rate
PD-1: Programmed Death 1
PD-L1: Programmed Death Ligand 1
SCCHN: Squamous cell carcinoma of the head and neck
SHP-1: Src Homology 2 domain Phosphatase 1
TGF-β: Transforming Growth Factor β
TIL: Tumor infiltrating lymphocytes
